# Enantiomeric Complexes Based on Ruthenium(III) and 2,2′-Biimidazole: X-ray Structure and Magnetic Properties

**DOI:** 10.3390/molecules28207213

**Published:** 2023-10-22

**Authors:** Marta Orts-Arroyo, Joel Monfort, Nicolás Moliner, José Martínez-Lillo

**Affiliations:** Instituto de Ciencia Molecular (ICMol)/Departament de Química Inorgànica, Universitat de València, c/Catedrático José Beltrán 2, Paterna, 46980 València, Spain; marta.orts-arroyo@uv.es (M.O.-A.); monrijo@alumni.uv.es (J.M.); fernando.moliner@uv.es (N.M.)

**Keywords:** ruthenium, 2,2′-biimidazole, enantiomers, crystal structures, crystal explorer, magnetic properties

## Abstract

We have prepared and characterized two Ru(III) compounds based on the 2,2′-biimidazole (H_2_biim) ligand, namely, a single complex of formula cis-[RuCl_2_(H_2_biim)_2_]Cl·4H_2_O (**1**) and a racemic mixture of formula {cis-[RuCl_2_(H_2_biim)_2_]Cl}_2_·4H_2_O (**2**), which contains 50% of Ru(III) complex **1**. Both compounds crystallize in the monoclinic system with space groups *C*2 and *P*2_1_ for **1** and **2**, respectively. These complexes exhibit the metal ion bonded to four nitrogen atoms from two H_2_biim molecules and two chloride ions, which balance part of the positive charges in a distorted octahedral geometry. Significant differences are observed in their crystal packing, which leads to the observation of differences in their respective magnetic behaviors. Despite having imidazole rings in both compounds, π–π stacking interactions occur only in the crystal structure of **2**, and the shortest intermolecular Ru···Ru separation in **2** is consequently shorter than that in **1**. Variable-temperature dc magnetic susceptibility measurements performed on polycrystalline samples of **1** and **2** reveal different magnetic behaviors at low temperatures: while **1** behaves pretty much as a magnetically isolated mononuclear Ru(III) complex with S = 1/2, **2** exhibits the behavior of an antiferromagnetically coupled system with S = 0 and a maximum in the magnetic susceptibility curve at approximately 3.0 K.

## 1. Introduction

The investigation of mononuclear ruthenium-based complexes has undergone huge advances in a wide range of research fields over the last two decades [1,2]. Indeed, recent ruthenium compounds, exhibiting promising properties, are emerging as suitable candidates for technological applications in research areas ranging from catalysis to chemotherapy [3,4,5,6,7,8,9,10,11,12,13].

Focusing on mononuclear Ru(II) and Ru(III) complexes, several systems have been specifically studied for their encouraging anticancer properties [14,15,16,17]. Many of them have been prepared with imidazole, indazole and other N- and S-donor ligands, such as the KP1019 and NAMI-A Ru(III)-based systems and, more recently, the TLD1433 Ru(II)-based system [18], which is obtained from a polypyridyl ligand [19,20,21]. All these mononuclear ruthenium-based complexes have emerged as promising candidates for obtaining anticancer compounds and are in fact the first Ru(II) and Ru(III) complexes to enter a human clinical trial [14,15,16,17]. Nevertheless, investigation of the magnetic properties of most of the mononuclear Ru(III) complexes remains scarcely explored. Indeed, there exists an important complexity to treating and investigating the experimental magnetic data of complexes based on this paramagnetic 4d metal ion, given that it exhibits a ^2^T_2g_ ground term with an important orbital contribution [22,23]. Nevertheless, the investigation of magnetic materials based on this 4d metal ion is ultimately very appealing given that the Ru(III) ion also displays more diffuse magnetic orbitals than those of 3d analogue compounds, which allow for intermolecular magnetic interactions that can also be conducted through space via weak forces in their crystal lattices and, in some cases, afford magnetic orderings in this way [24]. On the other hand, the study of pretty much isolated complexes of this paramagnetic 4d metal ion is also very appealing. In fact, only two mononuclear Ru(III) complexes displaying Single-Ion Magnet (SIM) behavior have been reported so far, which is fundamental to understanding the dynamics of the slow relaxation of the magnetization in Ru(III) complexes [22,23].

Concerning the choice of optimal organic ligands for the preparation of metal complexes that can exhibit additional interesting properties, 2,2′-biimidazole (H_2_biim) is a well-known ligand. It generates compounds displaying different protonation degrees with interesting supramolecular networks, and these are stabilized through hydrogen bonds and π–π stacking interactions [25,26,27,28,29,30]. In the literature, there are several works dealing with Ru(II) complexes based on the H_2_biim ligand, with these studies reporting on their redox [31], catalytic [32], luminescent [33,34,35] and cytotoxic [36,37] properties. However, the number of reported works based on Ru(III)–H_2_biim systems is significantly lower in comparison [38,39,40]. For all these reasons, we are very interested in exploring the coordination chemistry of mononuclear Ru(III) complexes based on H_2_biim.

**Figure 1:**
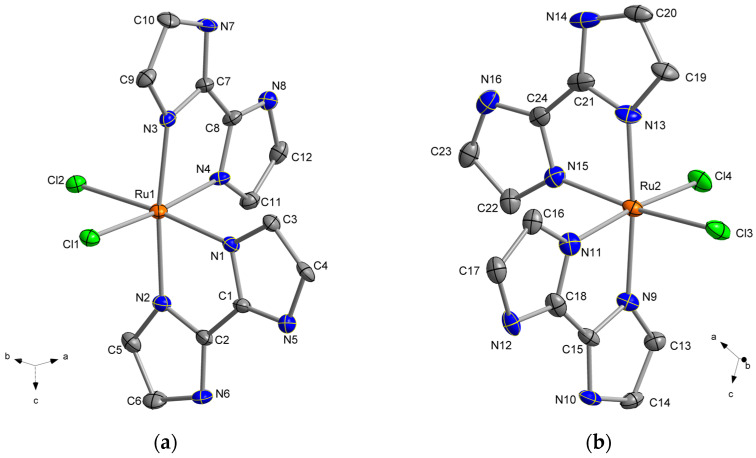
(**a**) View of the cationic Ru(III) complex in compound **1**; (**b**) view of one of the two enantiomeric units in compound **2**. H atoms, chloride counter-anions and H_2_O molecules have been omitted for clarity. Thermal ellipsoids are depicted at the 50% probability level.

Recently, we reported the in vitro anticancer activity of a racemic mixture of mononuclear Ru(III) complexes based on the H_2_biim ligand with a formula of {cis-[RuCl_2_(H_2_biim)_2_]Cl}_2_·4H_2_O (RUNAT-BI), which exhibits selective anticancer activity against highly aggressive cancer cell lines [41]. These results were previously patented. In this work, we report a comparative study of the crystal structure and magnetic properties of one of the enantiomers, cis-[RuCl_2_(H_2_biim)_2_]Cl·4H_2_O (**1**), together with the properties of the racemic mixture as a whole ({cis-[RuCl_2_(H_2_biim)_2_]Cl}_2_·4H_2_O (**2**)) (Figure 1).

## 2. Results and Discussion

### 2.1. Preparation of the Complexes

The ruthenium precursor RuCl_3_·H_2_O was made to react with the 2,2′-biimidazole ligand in a hydrochloric acid (3.0 M) solution at 90 °C, thus obtaining either the racemic mixture {cis-[RuCl_2_(H_2_biim)_2_]Cl}_2_·4H_2_O (**2**), when the Ru:H_2_biim ratio was 1:3, or only one of the two enantiomeric complexes, cis-[RuCl_2_(H_2_biim)_2_]Cl·4H_2_O (**1**), when the Ru:H_2_biim ratio was 1:1 (Figure 1). Isolation of the second enantiomeric complex was not achieved, maybe because of the different degrees of solubility of both species or because of other factors, and still remains challenging [41].

The synthetic process was carried out through a solvothermal reaction lasting 20.5 h. As a crystallization technique, a slow cooling process was performed for an additional 20.5 h until room temperature was reached. After that, crystals of **1** with a dark green color and crystals of **2** with a dark blue color were obtained with yields of approximately 45 and 30% for **1** and **2**, respectively. The crystals of **1** and **2** were suitable for X-ray diffraction data collection.

### 2.2. Description of the Crystal Structures

The crystal structures of **1** and **2** were investigated through single-crystal X-ray diffraction. Both compounds (**1** and **2**) crystallize in the monoclinic crystal system, with the non-centrosymmetric space group C2, whereas **2** crystallizes with the non-centrosymmetric space group P2_1_ (Table 1). Their crystal structures are made up of cationic [RuCl_2_(H_2_biim)_2_]^+^ complexes, chloride anions and H_2_O molecules (Figure 1). Compound **2** is a racemic mixture of two [RuCl_2_(H_2_biim)_2_]^+^ enantiomers and compound **1** crystallizes as only one of them, that is, **1** is an enantiopure species [42].

**Figure 2:**
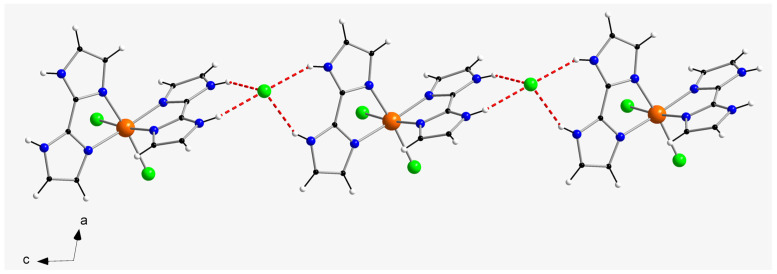
Detail of the one-dimensional motif generated by H-bonds (dashed red lines) connecting [RuCl_2_(H_2_biim)_2_]^+^ cations and chloride anions in **1**.

**Figure 3:**
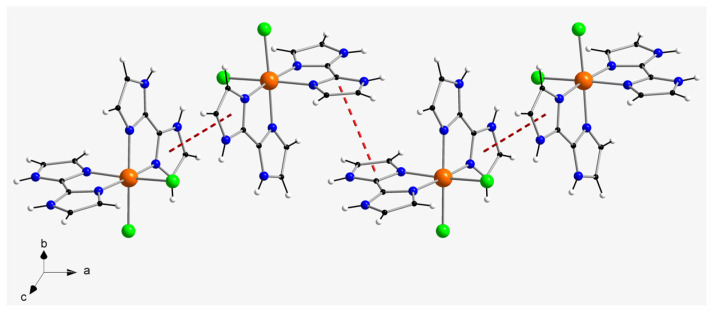
View along the crystallographic [011] direction of the one-dimensional motif generated by offset π–π stacking interactions in **2**.

In both compounds, each Ru(III) ion is bonded to two chloride ions and four nitrogen atoms of two 2,2′-biimidazole (H_2_biim) molecules, resulting in an octahedral geometry around the metal ion, which displays an important distortion in comparison with the regular one. The found average values of the Ru–N bond lengths are 2.050(1) Å for **1** and 2.058(1) Å for **2**, which are shorter than those of the Ru–Cl bond lengths with 2.367(1) Å for **1** and 2.351(1) Å for **2**. Furthermore, the Ru–N bond lengths in the trans position to the chloride anions are somewhat shorter than those in the cis position in both **1** and **2**. All these values are in agreement with those previously found in the crystal structures of similar Ru(III) complexes [43]. In **1**, the best equatorial plane around the metal ion is defined by the Cl1, Cl1a, N1 and N2 set of atoms, with N1 being shifted by 0.150(1) Å above this plane. In **2**, the best equatorial planes around Ru1 and Ru2 are established by the Cl1, Cl2, N3 and N7 and Cl3, Cl4, N11 and N15 sets of four atoms, respectively, and they form an angle of ca. 55.5(1)° between them. The organic molecule is planar in compounds **1** and **2**, with C−C and C−N bond length values that fall into the expected range found in similar complexes containing the 2,2′-biimidazole ligand as a neutral molecule [38,40] (Figure 1, Figure 2 and Figure 3).

**Figure 4:**
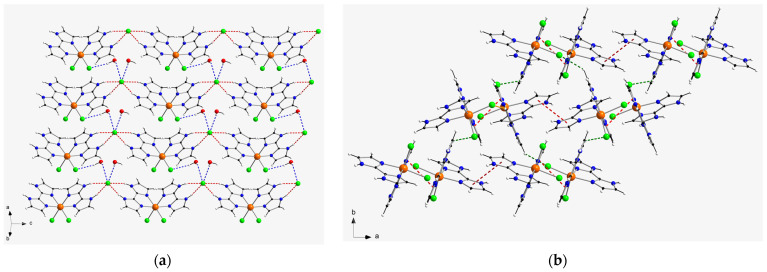
(**a**) View along the crystallographic [110] direction of the two-dimensional motif generated by H-bonds involving N–H groups and chloride ions (dashed red lines) and H_2_O molecules (only selected ones) and chloride ions (dashed blue lines) in **1**. (**b**) View along the crystallographic c axis of the two-dimensional arrangement of Ru(III) complexes connected through π–π stacking interactions (dashed red lines) and C-H···Cl interactions (dashed green lines) in **2**.

In the crystal packing of compound **1**, a one-dimensional motif is generated through hydrogen bonds affecting N–H groups of H_2_biim molecules and chloride counter-anions (Cl3···N5a and Cl3···N6a distances of 3.194(1) and 3.167(1) Å, respectively; (a) = x, y, z − 1), which connect the cations and anions and develop the chain formed along the c-axis direction (Figure 2). Additional H-bonding interactions between neighboring chains generate two-dimensional sheets, mainly through the Cl1···H_2_O(1w)···Cl3b pathway (O1w···Cl3b distance = 3.37(1) Å; (b) = x, y + 1, z + 1) (Figure 4). Despite having imidazole rings, no π–π stacking interactions take place in the crystal structure of **1**. Furthermore, the shortest intermolecular π···Cl distance is ca. 4.327 Å, which is too long to be considered a proper interaction. Thus, the shortest intermolecular Ru···Ru separation in **1** is ca. 7.15(1) Å and the shortest intermolecular Cl···Cl distance is approximately 5.62(1) Å. Finally, weak C–H···Cl contacts (average value of ca. 3.56(1) Å) hold together the two-dimensional sheets in the overall three-dimensional structure of **1**.

In the crystal packing of compound **2**, relatively short π–π stacking interactions of offset type (centroid–centroid distances vary in the range ca. 3.68–3.96 Å; (a) = −x+1, y − 1/2, −z+2) take place between imidazole rings of adjacent H_2_biim molecules and lead to the formation of cationic chains of [RuCl_2_(H_2_biim)_2_]^+^ complexes, which grow along the a-axis direction (Figure 3). Weak intermolecular C-H···Cl interactions (C4b···Cl2 distance = 3.66(1) Å; (b) = −x+2, y − 1/2, −z+2) connect the cationic chains and generate a two-dimensional supramolecular network with helical arrangement of the [RuCl_2_(H_2_biim)_2_]^+^ complexes in the crystal structure of **2** (Figure 4). The shortest intermolecular Ru···Ru distance in this compound is ca. 6.89(1) Å and the shortest intermolecular Cl···Cl distance is approximately 4.18(1) Å, which are shorter than those found for **1**. Additional intermolecular C-H···Cl (C···Cl distances covering the range ca. 3.49–3.68 Å) and π···Cl (ca. 3.90 Å) of interactions between [RuCl_2_(H_2_biim)_2_]^+^ complexes, along with H-bonds involving H_2_O molecules, stabilize the three-dimensional crystal structure in **2**.

### 2.3. Hirshfeld Surface Analysis

**Figure 5:**
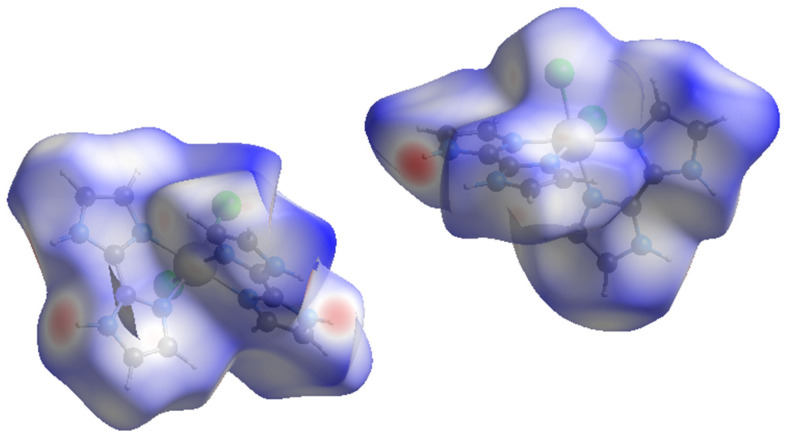
Hirshfeld surfaces mapped through d_norm_ function for the two enantiomeric units of compound **2**.

Hirshfeld surfaces of the cationic [RuCl_2_(H_2_biim)_2_]^+^ complexes in compounds **1** and **2** were calculated through the CrystalExplorer program, which is a program used for the surface analysis of molecules, as well as for the visualization and quantitative analysis of molecular crystals [44,45]. Thus, closer intermolecular interactions were analyzed by calculating surfaces that allow both a qualitative and quantitative visualization of the main intermolecular interactions detected in both compounds to be obtained. On these surfaces, the color red is assigned to the shorter contacts [44,45]. The nearest atoms outside (d_e_) and inside (d_i_) each surface direct the 3D mapping of these distances, considering at the same time a normalized contact distance (d_norm_) and taking into account some limitations generated by the atomic radii of the participating atoms [46,47,48]. The Hirshfeld surfaces for the H_2_biim-based complexes of **1** and **2** are given in Figure 5.

According to the CrystalExplorer data of **1**, the main intermolecular contacts involve the N–H groups of the H_2_biim ligands (Figure 5). The second most important interaction found on the Hirshfeld surface of **1** is that related to the Cl···H contacts involving chloride anions and N–H groups of neighboring mononuclear [RuCl_2_(H_2_biim)_2_]^+^ units, which covers ca. 29%. With regard to these latter interactions, approximately 14% come from the nearest atom outside the surface and the rest come from the nearest atom inside the surface.

In the case of compound **2**, the two enantiomeric complexes display very similar intermolecular interactions (Figure 5). In fact, they show the same fingerprint plot. In **2**, Cl···H interactions connecting chloride anions and C–H groups of adjacent [RuCl_2_(H_2_biim)_2_]^+^ complexes are the main intermolecular interaction (Figure 4), and comprise approximately 28% of the complete fingerprint plot of **2**. Finally, N···H contacts involving only N–H groups of neighboring imidazole rings are approximately 36% of the complete fingerprint plot of **2** (Figure 5).

### 2.4. Magnetic Properties

**Figure 6:**
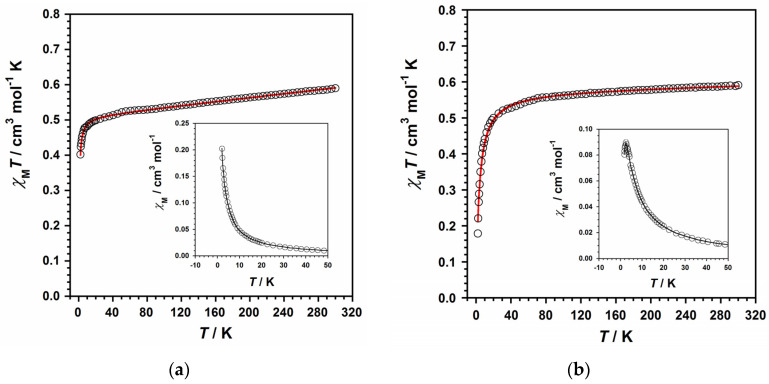
(**a**) The χ_M_*T* versus *T* curve for compound **1**. The inset displaying the χ_M_ versus *T* curve for **1**. (**b**) The χ_M_*T* versus *T* curve for compound **2**. The inset displaying the χ_M_ versus *T* curve for **2**. The solid red line is the best fit, whereas the solid black line is a guide for the eye.

The magnetic properties of compounds **1** and **2** were mainly studied using the data collected through direct current (dc) magnetic susceptibility measurements performed on the polycrystalline samples of both compounds. To keep these samples both immobilized and isolated during the measurements, the organic compound eicosene was used on them. The measurements were carried out through an external dc magnetic field of 0.5 T and covering the temperature range of 2–300 K. The χ_M_T versus T plots for compounds **1** and **2** (χ_M_ being the molar magnetic susceptibility per Ru(III) ion, with S = 1/2 and 4d^5^ configuration) are given in Figure 6. At room temperature, χ_M_T values for **1** and **2** were approximately 0.59 and 0.58 cm^3^mol^−1^K, respectively. These room-temperature values are quite similar to the ones reported in earlier works dealing with other mononuclear Ru(III) complexes exhibiting a low-spin configuration (t_2g_^5^) [49,50,51]. Upon cooling, χ_M_T values for **1** first constantly decrease with decreasing temperature to reach a value of ca. 0.49 cm^3^mol^−1^K at 15 K, and then more abruptly reach a final value of 0.40 cm^3^mol^−1^K at 2.0 K (Figure 6).

For **2**, χ_M_T values continuously decrease with decreasing temperature to a value of approximately 0.53 cm^3^mol^−1^K at 40 K. They subsequently continue decreasing faster, reaching a final value much lower than that of **1**: 0.18 cm^3^mol^−1^K at 2.0 K (Figure 6). This decrease in the χ_M_T values observed in both compounds at a medium–high range of temperature would account for the ^2^T_2g_ ground term of this paramagnetic metal ion, which exhibits spin–orbit coupling (SOC) and an important orbital contribution, as previously observed for mononuclear Ru(III) complexes [22,23]. Furthermore, a maximum of the magnetic susceptibility occurs at T_N_ ≈ 3.0 K in the χ_M_ versus T plot for **2** (see inset in Figure 6b), which would be assignable to an antiferromagnetically coupled system, indicating a zero spin value (S = 0) for **2**. In contrast, no maximum of the magnetic susceptibility is observed in the χ_M_ versus T plot for **1** (see inset in Figure 6a).

In the description of the crystal structures of both compounds, we have indicated that only compound **2** exhibits some singular short intermolecular interactions, such as π–π stacking contacts, which make the paramagnetic Ru(III) ions closer to each other in the crystal lattice of **2** in comparison with **1**. Thus, the observation of a maximum in the magnetic susceptibility curve would account for the relevance of this type of through-space interaction between Ru(III) ions, which has only been found in compound **2**, and not in **1**.

Taking these facts into consideration and in order to analyze the magnetic behavior of **1** and **2**, we have used the Hamiltonian of Equation (1) and its derived theoretical expression for magnetic susceptibility [52], including a θ term, to account for the detected intermolecular interactions. Furthermore, the three parameters in Equation (1), namely, energy gap (Δ), orbital reduction factor (κ) and the spin–orbit coupling constant (λ), are strongly correlated with each other (considering L = 1, S = 1/2 and g_ǀǀ_ = g_⊥_ = g for both **1** and **2**), as previously reported [22,52].

*Ĥ* = −*k**L**Ŝ +* Δ[***L****_Z_*^2^ − (1/3)***L****(**L*** + 1)] + *βH*(−*k**L*** + 2*Ŝ*) (1)

**Figure 7:**
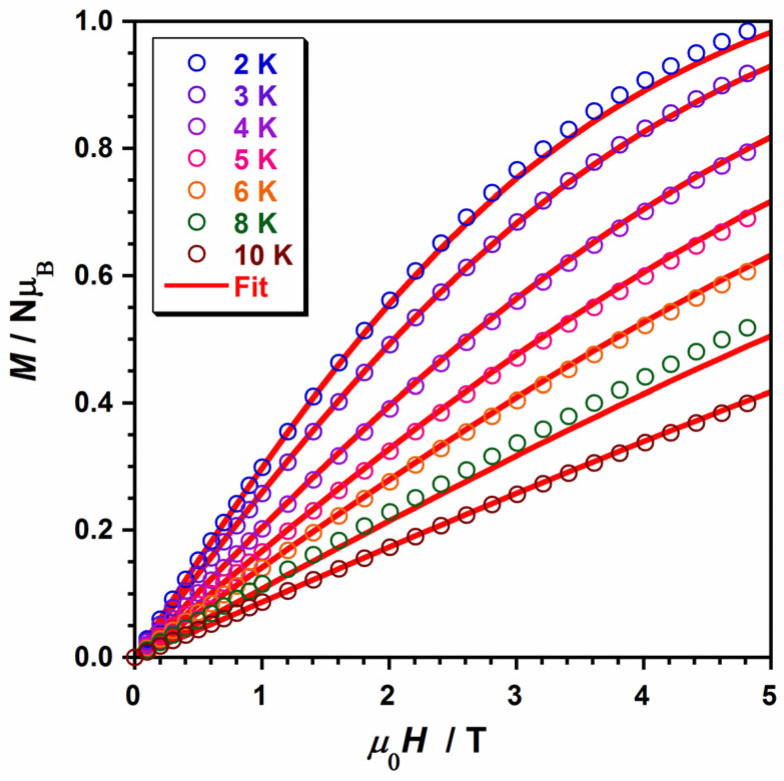
Plot of *M* versus *H* measured at several temperatures (2–10 K) for compound **1**. The solid red lines represent the best fit.

Indeed, we have reproduced the experimental magnetic data that draw the χ_M_T versus T curves of **1** and **2** by using the obtained values: Δ = 1057 cm^−1^, κ = 0.90, λ = −828 cm^−1^ and θ = −0.54 cm^−1^ for **1,** and Δ = 1679 cm^−1^, κ = 0.92, λ = −938 cm^−1^ and θ = −3.37 cm^−1^ for **2**. In addition, we used the PHI program to further study and compare the results of our fitted values [53], thus obtaining the following parameters: g = 2.42(1), θ = −0.50(1) cm^−1^ and TIP = 296 × 10^−6^ cm^3^mol^−1^ for **1** and g = 2.22(1), θ = −3.40(1) cm^−1^ and TIP = 12.8 − 10^−6^ cm^3^mol^−1^ for **2**, with the θ values being very close to the ones obtained through the former theoretical model. Next, these computed parameters were further employed to treat the field dependence of the molar magnetization (M versus H) curve, which is obtained at several temperatures and is given in Figure 7. This magnetization fitting process was not possible for **2**, given the strong correlation with antiferromagnetically coupling and the S = 0 of the system (Figure S1).

All these findings are consistent with the few magnetic parameters previously reported for some mononuclear Ru(III) complexes [22,23,49,50,51]. Finally, the results of the computed θ values would support the presence of antiferromagnetic exchange couplings for these compounds, with these being comparatively much more significant in the case of compound **2**, as expected, because of the magnetic susceptibility maximum observed for this compound [54].

## 3. Materials and Methods

### 3.1. Materials

All manipulations were performed under aerobic conditions, using the general chemicals as received. The ruthenium precursor RuCl_3_·H_2_O was acquired from Alfa Aesar (Haverhill, MA, USA) and the H_2_biim ligand was prepared following the procedure in the literature [55].

### 3.2. Preparation of the Complexes

#### 3.2.1. Synthesis of Compound **1**

Compound **1** was prepared through solvothermal synthesis between RuCl_3_·H_2_O (6.6 mg, 0.03 mmol) and 2,2′-biimidazole (4.1 mg, 0.03 mmol) in HCl (2.5 mL, 3.0 M) at 90 °C for 20.5 h, followed by a 20.5 h cooling process to room temperature. Subsequently, green plates of **1** were obtained and separated from a dark blue solution, and were suitable for X-ray diffraction data collection. Yield: ca. 45%. Anal. Calcd. for C_12_H_20_N_8_O_4_Cl_3_Ru (**1**): C, 26.3; H, 3.7; N, 20.5%. Found: C, 26.5; H, 3.4; N, 20.6%. ESI-MS (*m*/*z*): 441.83 (95.2%). Infrared (IR) peaks (sample prepared as KBr pellets): 3351 (m), 3275 (m), 3147 (m), 3129 (s), 3008 (m), 2923 (w), 2924 (m), 2764 (m), 1640 (s), 1543 (m), 1527 (s), 1417 (m), 1395 (m), 1320 (w), 1252 (w), 1178 (m), 1129 (m), 1078 (m), 1004 (w), 920 (m), 870 (w), 811 (w), 754 (s), 683 (m) and 517 (w) cm^−1^.

#### 3.2.2. Synthesis of Compound **2**

Compound **2** was prepared following the same procedure as **1** in HCl (2.5 mL, 3.0 M) but varying the amount of 2,2′-biimidazole (12.1 mg, 0.09 mmol). This reaction mixture was heated at 90 °C for 20.5 h and was then cooled down by means of a 20.5 h cooling process to room temperature; **2** crystallizes as dark blue crystals, which were isolated by filtration and were suitable for X-ray diffraction data collection. Yield: ca. 30%. Anal. Calcd. for C_12_H_16_N_8_O_2_Cl_3_Ru (**2**): C, 28.2; H, 3.2; N, 21.9%. Found: C, 28.5; H, 3.3; N, 22.2%. ESI-MS (*m*/*z*): 441.82 (94.6%). Infrared (IR) peaks (sample prepared as KBr pellets): 3278 (m), 3147 (m), 3129 (m), 3010 (m), 2923 (w), 2924 (m), 2765 (m), 1638 (s), 1526 (s), 1417 (m), 1394 (m), 1319 (w), 1252 (w), 1177 (m), 1129 (m), 1078 (m), 1008 (w), 922 (m), 870 (w), 811 (w), 754 (s), 682 (m) and 517 (w) cm^−1^.

### 3.3. X-ray Data Collection and Structure Refinement

X-ray diffraction data on single crystals with dimensions of 0.30 *×* 0.19 *×* 0.09 (**1**) and 0.45 *×* 0.13 *×* 0.09 mm^3^ (**2**) were collected on a Bruker D8 Venture diffractometer with a PHOTON II detector (Bruker, Mannheim, Germany) and by using monochromatized Mo-K_α_ radiation (λ = 0.71073 Å). Crystal parameters and refinement results for **1** and **2** are summarized in Table 1. The structures were solved by standard direct methods and subsequently completed by Fourier recycling using the SHELXTL (SHELXTL-2013/4) [56] software packages and refined by the full-matrix least-squares refinements based on F^2^ with all observed reflections. Compound **2** reveals the pseudosymmetric space group P2_1_/n, but this symmetry breaks due to the slightly different positions of the counterions and water molecules, which lack hydrogen atoms. The final graphical manipulations were performed with the DIAMOND program [57]. The CCDC Deposition Numbers are 2286941 and 2286942 for **1** and **2**, respectively.

### 3.4. Physical Measurements

Elemental analyses of C, H and N elements were performed by means of an elemental analyzer (CE Instruments CHNS1100, LBIP Ltd., Lichfield, UK) and electrospray ionization mass (ESI-MS) analyses were performed through a SCIEX TripleTOF 6600+ (DH Technologies Development Pte Ltd., Singapore) mass spectrometer (by using a direct infusion electrospray ionization source), which are located in the Central Service for the Support of Experimental Research (SCSIE) at the University of Valencia. The infrared spectra (IR) of **1** and **2** were recorded with a PerkinElmer Spectrum 65 FT-IR spectrometer (PerkinElmer, Inc., Waltham, MA, USA) in the 4000–400 cm*^−^*^1^ region. A spectral resolution of 4 cm^−1^ with 25 scans for each spectrum was used. Dc magnetic susceptibility measurements, of variable-temperature type and with solid samples, were collected on a Quantum Design MPMS-XL SQUID magnetometer (Louisiana State University (LSU), Baton Rouge, LA, USA), which was equipped with a 5 T dc magnet in the Institute of Molecular Science (ICMol) at the University of Valencia. The diamagnetic contributions of the experimental magnetic data were corrected for both the sample holder and the eicosene used in the samples of **1** and **2**. Finally, the diamagnetic contribution of the involved atoms was corrected for both compounds using tabulated Pascal’s constants [58].

## 4. Conclusions

In conclusion, we have reported on the synthesis and the crystallographic and magnetic studies of two enatiomeric Ru(III) compounds obtained with the 2,2′-biimidazole ligand. They crystallize as an enantiopure complex with formula cis-[RuCl_2_(H_2_biim)_2_]Cl·4H_2_O (**1**), and hence in a non-centrosymmetric space group (C2 space group) (as expected [37]), and as a racemic mixture, {cis-[RuCl_2_(H_2_biim)_2_]Cl}_2_·4H_2_O (**2**) (P2_1_ space group), hence containing 50% of the Ru(III) complex **1**. The in vitro anticancer properties of **1** and **2** were earlier reported; while **2** demonstrated selectivity between tumor and non-tumor cell lines and increased proapoptotic gene expression, **1** did not show any similar effect. These results have previously been patented [36].

Despite their great structural similarities, there are important crystallographic differences between the reported crystal packings of both structures. In their crystal lattice, there are only intermolecular π–π stacking interactions present in **2**. This fact makes the shortest intermolecular Ru···Ru distance in **2** shorter than that of **1**. Furthermore, a complete study on the Hirshfeld surfaces of the cationic [RuCl_2_(H_2_biim)_2_]^+^ units in compounds **1** and **2**, performed through the CrystalExplorer program, shows that intermolecular interactions connecting chloride anions and C–H groups of adjacent [RuCl_2_(H_2_biim)_2_]^+^ units are present in both compounds. Finally, we have observed that these crystallographic features have a significant impact on their magnetic behavior. Indeed, the study of the magnetic properties of **1** and **2** by means of dc susceptibility measurements gave us a θ value for **2** that is much higher than that obtained for **1**. In addition, we only observed a maximum in the magnetic susceptibility versus temperature curve for **2**. Hence, in these Ru(III) compounds, the different dispositions of the cationic [RuCl_2_(H_2_biim)_2_]^+^ complexes in their crystal lattices play a crucial role in determining the structure–property relationship. We are now working on other halide Ru(III) compounds based on N-donor ligands. This work is still in progress.

## 5. Patents

Compound **2** has been certified as an international patent with certificate PCT/ES2022/070415, Universitat de València and Fundación INCLIVA (2021): Ruthenium-biimidazole compound (RUNAT-BI) and its therapeutic use.

## Figures and Tables

**Table 1:** Summary of the crystal data and structure refinement parameters for **1** and **2**.

Compound	1	2
CIF	2286941	2286942
Formula	C_12_H_12_N_8_O_4_Cl_3_Ru	C_24_H_24_N_16_O_4_Cl_6_Ru_2_
Fw/g mol^−1^	539.72	1015.43
Temperature/K	120(2)	120(2)
Crystal system	monoclinic	monoclinic
Space group	*C*2	*P*2_1_
*a*/Å	7.199(1)	13.457(1)
*b*/Å	12.342(1)	11.317(1)
*c*/Å	11.571(1)	13.749(1)
α/°	90	90
β/°	103.28(1)	115.56(1)
γ/°	90	90
*V*/Å^3^	1000.52(14)	1889.10(1)
*Z*	2	2
*D*_c_/g cm^−3^	1.792	1.785
μ(Mo-K_α_)/mm^−1^	1.221	1.279
*F*(000)	534	1004
Goodness-of-fit on *F*^2^	1.126	1.060
*R*_1_ [*I* > 2σ(*I*)]/all data	0.0448/0.0516	0.0413/0.0420
w*R*_2_ [*I* > 2σ(*I*)]/all data	0.0859/0.0899	0.1269/0.1281
Abs. structure (Flack)	0.01(2)	0.50(2)

## Data Availability

The data provided in this investigation are available from the corresponding author on reasonable request.

## Supplementary Materials

The following supporting information can be downloaded at: https://www.mdpi.com/article/10.3390/molecules28207213/s1, Figure S1 (Plot of *M* vs*. H* measured at 2.0 K for compound **2**). CIF file of **1**. CIF file of **2**.
