# Supplementary material for: Enantiomeric Complexes Based on Ruthenium(III) and 2,2′-Biimidazole: X-ray Structure and Magnetic Properties
Source: Molecules. 2023 Oct 22;28(20):7213. doi: 10.3390/molecules28207213 (PMC10609436; doi:10.3390/molecules28207213)
Supplement: Supplementary file 1 [file molecules-28-07213-s001.zip › molecules-2576878-supplementary/Revised_SI.pdf]

Supplementary Information (SI)

Enantiomeric Complexes Based on Ruthenium(III) and 2,2 -Biimidazole: X-ray Structure and Magnetic Properties

Marta Orts-Arroyo , Joel Monfort, Nicolás Moliner and José Martínez-Lillo *

Instituto de Ciencia Molecular (ICMol)/Departament de Química Inorgànica, Universitat de València, c/Catedrático José Beltrán 2, Paterna, 46980 València, Spain; marta.orts-arroyo@uv.es (M.O.-A.); monrijo@alumni.uv.es (J.M.); fernando.moliner@uv.es (N.M.)
* Correspondence: f.jose.martinez@uv.es; Tel.: +34-9635-44460

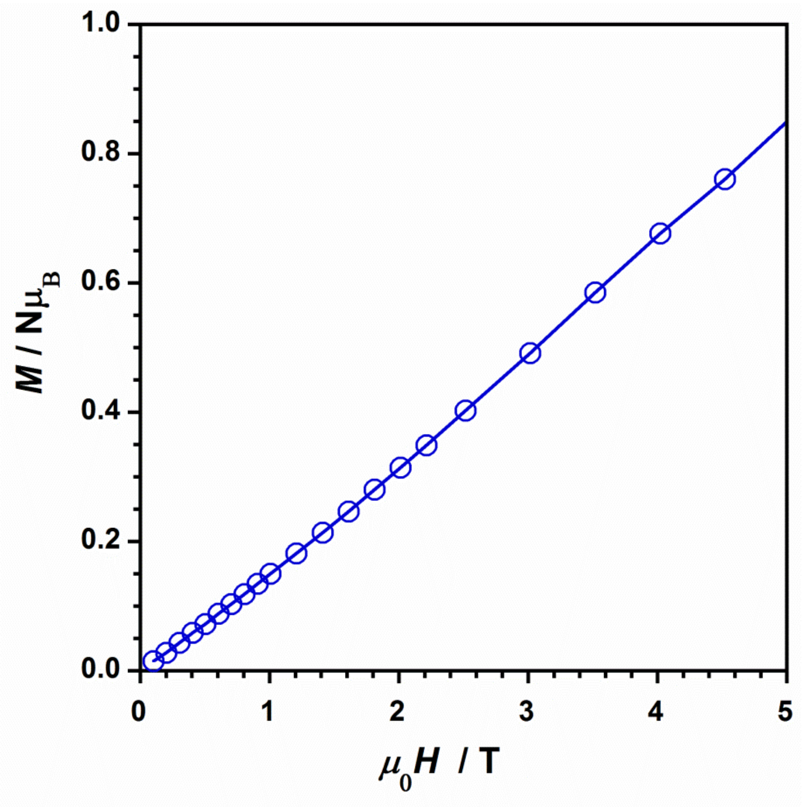

Figure S1. Plot of *M versus H* measured at 2.0 K for compound **2**. The solid blue line represents just a guide for the eye.
